# One Bout of Aerobic Exercise Can Enhance the Expression of *Nr1d1* in Oxidative Skeletal Muscle Samples

**DOI:** 10.3389/fphys.2021.626096

**Published:** 2021-02-01

**Authors:** Rafael L. Rovina, Alisson L. da Rocha, Bruno B. Marafon, José R. Pauli, Leandro P. de Moura, Dennys E. Cintra, Eduardo R. Ropelle, Adelino S. R. da Silva

**Affiliations:** ^1^School of Physical Education and Sport of Ribeirão Preto, University of São Paulo (USP), Ribeirão Preto, Brazil; ^2^Postgraduate Program in Rehabilitation and Functional Performance, Ribeirão Preto Medical School, University of São Paulo (USP), Ribeirão Preto, Brazil; ^3^Laboratory of Molecular Biology of Exercise (LaBMEx), School of Applied Sciences, University of Campinas (UNICAMP), Limeira, Brazil

**Keywords:** *Nr1d1*, exercise, skeletal muscle, atrophy, mitochondrial biogenesis

## Abstract

The nuclear receptor subfamily 1, group D member 1 (*Nr1d1)*, plays a role in the skeletal muscle’s oxidative capacity, mitochondrial biogenesis, atrophy genes, and muscle fiber size. In light of the effects of physical exercise, the present study investigates the acute response of *Nr1d1* and genes related to atrophy and mitochondrial biogenesis on endurance and resistance exercise protocols. In this investigation, we observed, after one bout of endurance exercise, an upregulation of *Nr1d1* in soleus muscle, but not in the gastrocnemius, and some genes related to mitochondrial biogenesis and atrophy were enhanced as well. Also, analysis of muscle transcripts from diverse isogenic BXD mice families revealed that the strains with higher *Nr1d1* gene expression displayed upregulation of AMPK signaling and mitochondrial-related genes. In summary, a single session of endurance exercise can enhance the *Nr1d1* mRNA levels in an oxidative muscle.

## Introduction

Acute and chronic physical exercises can be used as a non-pharmacological strategy to prevent and treat several diseases, improve life quality, and maximize athletes’ performance (Gleeson et al., 2011). The acute resistance exercise can stimulate the gain of muscle mass and strength through the activation of protein kinase B (Akt)/mammalian target of rapamycin (mTOR) pathway (Bodine et al., 2001). On the other hand, glucocorticoids can inhibit the mTOR pathway through Krüppel-like factor 15 (KLF15). This pathway has an important function in skeletal muscle catabolism due to the transcriptional upregulations of atrogin-1 and muscle RING-finger protein-1, which are related to muscle mass control. Also, branched-chain amino acid transaminase 2 (BCAT2) is a regulator of mTOR activity, but through the participation in the BCAA catabolism event (Liu et al., 2017; Shimizu et al., 2011).

The acute endurance exercise is related to the activation of the AMP-activated protein kinase (AMPK)/proliferator-activated receptor gamma coactivator 1-alpha (PGC1-α) pathway, increasing skeletal muscle oxidative capacity, mitochondrial content, and maximal oxygen uptake (VO2_max_) (Hardie et al., 2012). The PGC1-α is classically known as a transcriptional coregulator of events like mitochondrial biogenesis and angiogenesis, playing a central role in endurance adaptations. However, as Ruas and colleagues (Woldt et al., 2013) described, the isoform named PGC1-α4 is linked to hypertrophy responses in skeletal muscle. This specific isoform of PGC-1 regulates the insulin-like growth factor 1 and myostatin pathways, having a different role from the other isoforms.

In an elegant investigation, Woldt et al. (2013) showed that the knocking down of the nuclear receptor subfamily 1, group D member 1 (*Nr1d1*), the codify gene of the protein Rev-erb-alpha, was linked to lower skeletal muscle oxidative capacity, mitochondrial content, and VO_2_max. Interestingly, the Rev-erb-alpha can regulate the mitochondrial biogenesis through the Stk11 (Serine/threonine kinase 11)-Ampk-Sirt1-Ppargc1α pathway. The Stk11 plays a significant role in regulating the expression of Ampk and, consequently, of the Ppargc1α. On the other hand, a deficiency of Rev-erb-alpha leads to lower Ppargc1α expression in skeletal muscle, one of the central genes in the mitochondrial biogenesis pathway (Woldt et al., 2013). Also, Mayeuf-Louchart et al. (2017) demonstrated that the genetic ablation of *Nr1d1* was associated with the increased expression of the atrophy-related genes and reduced muscle mass and fiber size in skeletal muscle samples.

Knowing the pharmacological activation of Rev-erb-alpha enhanced mitochondrial content and respiration in skeletal muscle cells, as well as exercise capacity, and reverted the negative effects of dexamethasone treatment in skeletal muscle mass and atrophy-related genes (Woldt et al., 2013; Mayeuf-Louchart et al., 2017), it is relevant to investigate physiological strategies capable of increasing the expression of this gene in skeletal muscle. Therefore, we investigated the acute effects of resistance and endurance exercise protocols on the messenger ribonucleic acid (mRNA) levels of *Nr1d1*, *Prkaa1*, *Ppargc1a*, *Fbxo32*, *Trim63*, *Ubc*, and *Bcat2* in mice skeletal muscle samples.

## Materials and Methods

### Experiment Animals

Eight-week-old C57BL/6 mice from the Central Animal Facility of the Ribeirão Preto campus from the University of São Paulo (USP) were used for the experiment. The animals were accommodated in sterile micro-insulators (three animals per cage) in a ventilated rack (INSIGHT^®^, Ribeirão Preto, Brazil) with controlled temperature (22 ± 2°C) on a 12:12-h light-dark inverted cycle. Food (Purina chow) and water were provided *ad libitum*. According to the Brazilian College of Animal Experimentation (COBEA), all experimental procedures were approved by the Ethics Committee of the University of São Paulo (I.D. 2017.5.33.9037).

**FIGURE 1:**
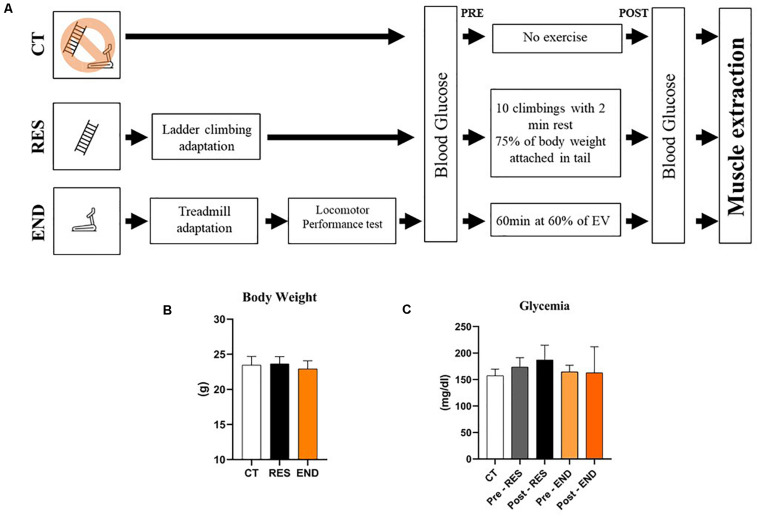
Schematic representation of the experimental procedures **(A)**; Body weight of the CT, RES, and END groups **(B)**; Glycemia of the CT, RES, and END groups **(C)**. Data correspond to the mean ± SD of *n* = 5 mice/group. CT, sedentary mice; RES, mice submitted to the resistance protocol; END, mice submitted to the endurance protocol.

Mice were divided into three experimental groups: Control (CT; sedentary), Resistance (RES; submitted to the resistance exercise protocol), and Endurance (END; submitted to the endurance exercise protocol). The sample size (*n*) for each experiment is available in the figure legends. Mice from the RES group were submitted to 5 days of adaptation on a ladder-climbing (INSIGHT^TM^, Ribeirão Preto, Brazil) with and without external load (Wang et al., 2016). The ladder had 1,110 mm of height, 80° of inclination, and 85 steps with a distance of 6 mm between each. Also, mice from the END group were submitted to 5 days of adaptation on a treadmill (INSIGHT^TM^, Ribeirão Preto, Brazil), 10 min/day, at a speed of 6 m.min^–1^. The experimental design is described in Figure 1A.

### Resistance Group

Mice from the RES group were submitted to 5 days of adaptation on a ladder-climbing (INSIGHT^TM^, Ribeirão Preto, Brazil) without external load. The ladder had 1,110 mm of height, 80° of inclination, and 85 steps with a distance of 6 mm between each. On the day of the acute exercise protocol, the RES group first performed one climb without external load to warm-up. After that, an external load corresponding to 75% of body weight was applied at the base of each animal’s tail, and mice performed ten climbs with a 2 min recovery between each. This protocol was adapted from the investigation of Wang and coworkers (Wang et al., 2015), which verified molecular signs of hypertrophy in rodents. The total duration of the acute resistance exercise protocol was approximately 20 min.

### Endurance Group

Mice from the END group were submitted to 5 days of adaptation on a treadmill (INSIGHT^TM^, Ribeirão Preto, SP, Brazil), 10 min/day, at a speed of 6 m.min^–1^. After 48 h of the treadmill adaptation, the locomotor performance test started at an initial velocity of 6m/min, at 0% of inclination for the END group, with 3 m/min increments every 3 min until voluntary exhaustion. The exhaustion velocity (EV) was used to prescribe the exercise intensity for the END group. Mice ran at 60% of the EV at 0% inclination for 60 min (Ferreira et al., 2007). Previously, Ferreira et al. (2007) showed the intensity corresponding to 60% of EV obtained in the incremental load test was similar to the MLSS intensity, which can be defined as the highest exercise intensity in which balance between the production and removal of blood lactate occurs, and is used as the gold standard to determine exercise intensity (Da Silva et al., 2010; Ferreira et al., 2007).

### Glucose Levels

The blood from the tail tip was collected, and glucose levels were measured before and immediately after the acute physical exercise protocols using a glycemic monitoring system (Accu-Chek^TM^ Active model, Roche, Santo André, Brazil).

### Extraction of the Skeletal Muscle

Immediately after the acute physical exercise protocols, the animals were anesthetized by an intraperitoneal administration of xylazine (10 mg/kg of body weight) and ketamine (100 mg/kg of body weight). As soon as the loss of pedal reflexes confirmed the effect of anesthesia (i.e., about 5 min), the gastrocnemius and soleus samples were removed, washed with sterile saline, and stored for reverse transcription-quantitative polymerase chain reaction (RTq-PCR) technique [storage at −80°C with RNAlater (Ambion, Life Technologies, Grand Island, NY, United States)].

### Reverse Transcription-Quantitative Polymerase Chain Reaction

Total RNA from the whole gastrocnemius and soleus were extracted with TRIZOL (Invitrogen, Carlsbad, CA, United States). All procedures were performed under standard RNase-free conditions to avoid exogenous RNase contamination. The quantitative Real time-PCR technique was performed by the ViiA7 Real-Time PCR System (Applied Biosystems) for analysis of gene mRNA expression for *Nr1d1* (nuclear receptor subfamily 1, group D, member 1), *Prkaa1* (protein kinase, AMP-activated, alpha 1 catalytic subunit), *Ppargc1a* (peroxisome proliferative activated receptor, gamma, coactivator 1 alpha), *Mtor* (mechanistic target of rapamycin kinase), *Fbxo32* (F-box protein 32), *Trim63* (tripartite motif-containing 63), *Ubc* (ubiquitin C), and *Bcat2* (branched-chain aminotransferase 2, mitochondrial).

Reverse transcription-quantitative polymerase chain reaction was performed in duplicate with the following reagents: 5 μl HOT FIREPol EvaGreen qPCR SuperMix from Solis BioDyne (Tartu, Estonia), 1 μl primer forward, 1 μl primer reverse (both at a final concentration of 200 nM), 1 μl cDNA (10 ng), and 1 μl d H_2_O. Each amplification reaction occurred with standard cycling with the following cycles: one cycle at 95°C for 12 min, 40 cycles of 15 s at 95°C, 25 s at 60°C, and 25 s at 72°C. Relative quantitation was calculated by the 2^–ΔΔCT^ method using Thermo Fisher Cloud Software, RQ version 3.7 (Life Technologies Corporation, Carlsbad, CA, United States). All values were corrected by the value obtained for the *Gapdh* (Glyceraldehyde-3-phosphate dehydrogenase) amplification. Primer designs are described in Table 1.

**TABLE 1:** The primers design.

Gene	Forward	Reverse
*Nr1d1*	AGAGAGGCCATCACAACCTC	TGTAGGTGATAACACCACCTGT
*Prkaa1*	CCAGGTCATCAGTACACCATCT	TTTCCTTTTCGTCCAACCTTCC
*Ppargc1a*	GAGTTGAAAAAGCTTGACTGGC	CAGCACACTCTATGTCACTCC
*Mtor*	CCACGTGGTTAGCCAGACT	TAGCGGATATCAGGGTCAGGA
*Fbxo32*	CAAAGGAAGTACGAAGGAGCG	TCAGCTCCAACAGCCTTACTA
*Murf1*	CAGGCTGCGAATCCCTACTG	GCCGGTCCATGATCACTTCA
*Ubc*	CGCGCTGATCCCTCCG	CTGCATCGTCTCTCTCACGG
*Bcat2*	TATGGACCCACTGTGGCTGT	CAGCTCCAGTACTCCGTCTTC
*Gapdh*	AAGAGGGATGCTGCCCTTAC	CGGGACGAGGAAACACTCTC

**Nr1d1*, Nuclear receptor subfamily 1, group D, member 1; *Prkaa1*, Protein kinase, AMP-activated, alpha 1 catalytic subunit; *Ppargc1a*, Peroxisome proliferative activated receptor, gamma, coactivator 1 alpha; *Mtor*, Mechanistic target of rapamycin kinase; *Fbxo32*, F-box protein 32; *Trim63*, Tripartite motif-containing 63; *Ubc*, Ubiquitin C; *Bcat2*, Branched-chain aminotransferase 2, mitochondrial; *Gapdh*, Glyceraldehyde-3-phosphate dehydrogenase.*

### Bioinformatic Analysis

Correlation analyses were performed using a data set from muscle *Nr1d1* [EPFL/LISP BXD CD Muscle Affy Mouse Gene 1.0 ST (Dec11) RMA] and muscle genes related to the AMPK signaling and mitochondria of genetically diverse BXD mice as previously published (Andreux et al., 2012). The four strains with the highest *Nr1d1* values and the four strains with the lowest *Nr1d1* values were selected to correlate with the other genes. All data are accessible on Genetwork^1^. The heatmap graph was obtained using the Gene-E software.

### Statistical Analysis

Results are expressed as mean ± standard deviation (SD). The Shapiro–Wilk’s W-test was used to verify data normality, and Levene’s test was used to test the homogeneity of variances. One-way ANOVA and *post hoc* and Bonferroni were used to verify the effects of acute experimental protocols. When applicable, Pearson’s correlation coefficient was used to test the association between the studied parameters. All statistical analyses were two-sided, and the significance level was set at *p* ≤ 0.05. Statistical analyses were performed using the software SPSS v.20.0 for Windows (IBM, Chicago, IL, United States).

## Results

### Metabolic Parameters and Gene Expression

**FIGURE 2:**
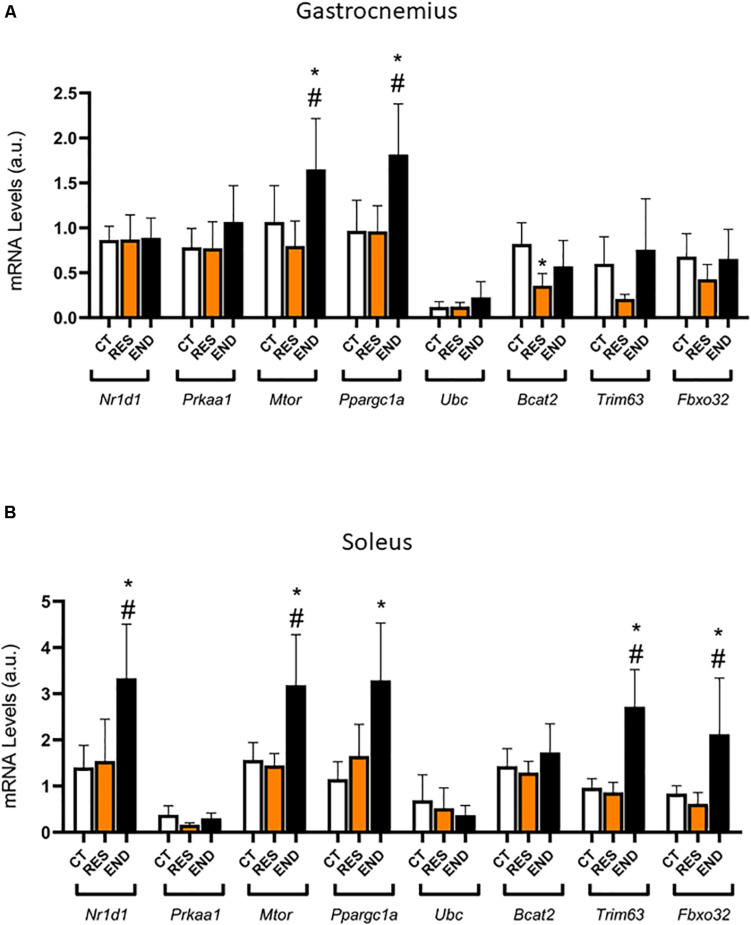
**(A)** Gastrocnemius mRNA levels of *Nr1d1*, *Prkaa1*, *Ppargc1a*, *Mtor*, *Fbxo32*, *Trim63*, *Ubc*, and *Bcat2*. **(B)** Soleus mRNA levels of *Nr1d1*, *Prkaa1*, *Ppargc1a*, *Mtor*, *Fbxo32*, *Trim63*, *Ubc*, and *Bcat2*. Data correspond to the mean ± SD of *n* = 5 mice/group. *p ≤ 0.05 *vs.* END group; ^#^*p* ≤ 0.05 *vs.* RES group. CT, sedentary mice; RES, mice submitted to the resistance protocol; END, mice submitted to the endurance protocol.

Figures 1B,C show body weight and glycemia were not different between the experimental groups, respectively. For the gastrocnemius samples, the mRNA levels of *Nr1d1*, *Prkaa1*, *Fbxo32*, *Murf*, and *Ubc* were not different between the experimental groups (Figure 2A). The mRNA levels of *Ppargc1a* and *Mtor* were higher for the END group than the CT and RES groups (Figure 2A). Also, the mRNA levels of *Bcat2* were lower for the RES group compared to the CT group (Figure 2A).

For the soleus samples, the mRNA levels of *Nr1d1, Mtor, Fbxo32*, and *Trim63* were higher for the END group than the CT and RES groups (Figure 2B). Also, the mRNA levels of *Ppargc1a* were higher for the END group compared to the CT group (Figure 2B). The mRNA levels of *Prkaa1*, *Ubc*, and *Bcat2* were not different between the experimental groups (Figure 2B).

**FIGURE 3:**
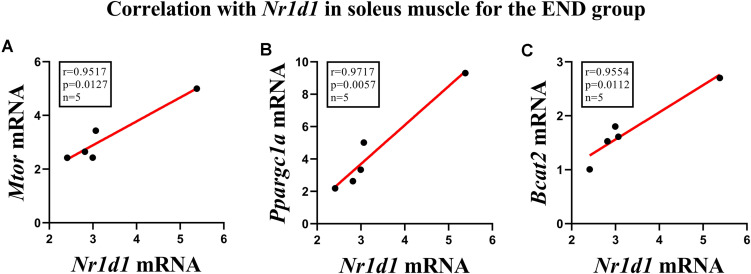
Correlations of *Nr1d1* with **(A)** *Mtor*, **(B)** *Ppargc1a*, and **(C)** *Bcat2* in soleus muscle for the endurance group.

Figure 3 shows the correlation between the responses of *Nr1d1* gene in the soleus muscle for the END group, where *Mtor* (*p* < 0.01; *r* = 0.95), *Ppargc1a* (*p* < 0.005; *r* = 0.97) and *Bcat2* (*p* < 0,01; *r* = 0.95) displayed significant positive correlations. The *Nr1d1* responses for the RES group in the soleus muscle did not present significant correlations with the other genes. The *Nr1d1* responses in gastrocnemius for both RES and END groups were not significantly correlated with other genes.

### Bioinformatic Results

**FIGURE 4:**
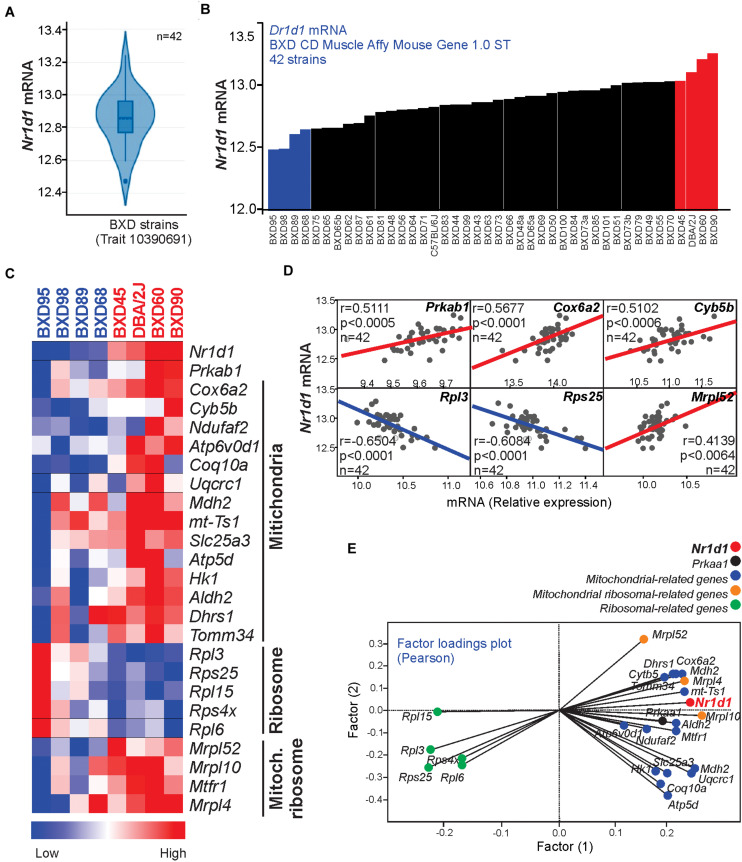
**(A)** The violin plot shows the *Nr1d1* gene expression distribution in the muscle of 42 BXD mice strains **(B)** *Nr1d1* gene expression in each BXD strain muscle. **(C)** Heatmap graph highlighting the gene expression in 4 BXD strains with low (blue) and 4 BXD strains with high (red). **(D)** Pearson’s correlation and **(E)** two factors analysis graphs show the correlation between *Nr1d1*gene expression and *Prkaa1* mRNA levels, mitochondrial-related, and ribosomal-related genes. Blue lines indicate negative and red lines indicate a positive correlation.

To test our hypothesis in different mice strains, we resorted to a bioinformatics analysis using the BXD database, which is the largest and best-categorized family of isogenic strains and provides a broad set of data appropriate for investigations. First, we visualized the distribution of muscle *Nr1d1* mRNA levels in 42 strains of isogenic BXD mice, highlighting four strains with lower (BXD95, 98, 89, and 68) and four strains with higher (BXD45, DBA/2J, BXD60, and BXD90) levels of *Nr1d1* mRNA in the skeletal muscle (Figures 4A,B). The transcriptomic analysis demonstrated that the variations of *Nr1d1* influenced several genes related to AMPK signaling and mitochondria (Figures 4C–E).

## Discussion

**FIGURE 5:**
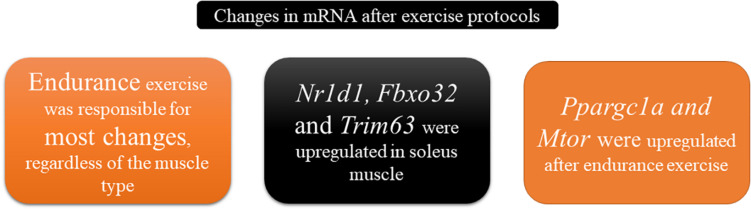
Schematic representation summarizing the main findings of the present investigation.

The control of the muscular physiological state during rest or exercise requires fine adjustment between the oxidative capacity of fibers with the consequent use of energetic substrates and molecular signals dependent on regulated genomic orchestration. Therefore, the main findings of this study were: (1) Regardless of the skeletal muscle type, most of the significant changes were observed for the END group; (2) While the mRNA levels of *Ppargc1a* and *Mtor* were upregulated in both skeletal muscle samples after the END protocol, the mRNA levels of *Nr1d1*, *Fbxo32*, and *Trim63* were upregulated only in the predominant oxidative skeletal muscle; (3) The mRNA levels of *Bcat2* were downregulated in the gastrocnemius sample after the RES protocol. Figure 5 summarizes the data of the present investigation.

BXD mice population has been extensively used to explore the causal and mechanistic links between genomes and several physiological or pathological conditions (Andreux et al., 2012; Wu et al., 2014; Williams et al., 2016). Here, we found a differential distribution of *Nr1d1* gene expression in skeletal muscle from 42 BXD strains. Four families with higher *Nr1d1* gene expression displayed upregulation of AMPK signaling and mitochondrial-related genes. These data reinforce previous results showing *Nr1d1* activation led to an increase of mitochondrial content and respiration, as well as exercise capacity (Woldt et al., 2013).

Except for the *Bcat2* mRNA levels, the RES protocol did not modulate the other genes in any of the skeletal muscle samples, which can be linked to the lower volume of the RES protocol (approximately 20 min) compared to the END protocol (60 min). The global deletion of *Bcat2* led to elevated gastrocnemius protein turnover in mice (Lynch et al., 2015). Our RES protocol reduced the mRNA levels of *Bcat2* in the gastrocnemius muscle. In contrast, Roberson et al. (2018) did not observe significant changes for the *Bcat2* in the vastus lateralis of subjects performing ten sets of five repetitions of a back squat exercise at 80% of one-repetition maximum. Possibly, the main differences between Roberson’s investigation (Roberson et al., 2018) and ours are the experimental models (humans vs. rodents), tissue extraction times (2 h after vs. immediately after), and training state (trained subjects vs. sedentary mice).

The exercise intensity and volume may justify that most alterations were observed for the END group, which ran for 60 min at 60% of the EV that corresponds to the moderate-exercise intensity (Ferreira et al., 2007; Da Silva et al., 2010). Interestingly, the END protocol increased the mRNA levels of Ppargc*1a* and *Mtor* in both skeletal muscle samples. Transgenic mice overexpression *Ppargc1a* highlighted its fundamental role in regulating the mitochondrial biogenesis in skeletal muscles (Calvo et al., 2008). Also, the sensitivity of *Ppargc1a* to acute endurance exercise was recently reviewed (Popov, 2018).

On the other hand, mTOR complex 1 inhibition prevents growth and increases atrophy in skeletal muscles (Bentzinger et al., 2013). Corroborating our findings, Hayasaka et al. (2014) verified an increase of mTOR phosphorylation in serine 2,448 immediately after a treadmill running at 28 m/min for 60 min in gastrocnemius muscles of Sprague–Dawley rats. Woldt and coauthors (Woldt et al., 2013) demonstrated the higher expression of Rev-erb-alpha occurred in soleus (a predominant oxidative muscle), and its global deletion impaired mitochondrial and exercise capacity. Also, these authors verified that 8 weeks of treadmill running at an initial intensity of 8 m/min, which increased to 16 m/min during the last 4 weeks, 5 days a week, 1–2 h per day, increased the protein contents of Rev-erb-alpha in gastrocnemius and soleus samples (Woldt et al., 2013).

Although Yasumoto et al. (2015) verified an increased expression of *Nr1d1* in mice’s skeletal muscle in response to chronic wheel-running activity, to the best of our knowledge, this is the first investigation showing the *Nr1d1* was elevated immediately after one single bout of moderate-intensity endurance exercise. Interestingly, this result occurred only in the most oxidative skeletal muscle. Although we did not find significant changes after the RES protocol, future studies should evaluate different time-points since the mRNA expression alterations can occur up to 12 h after acute exercise (Egan and Zierath, 2013). Atrogin-1 and MuRF1 are encoded by the atrophy-related genes *Fbxo32* and *Trim63*, respectively (Dang et al., 2016). The Nr1d1 and the mRNA levels of *Fbxo32* and *Trim63* in soleus were elevated after the END protocol. These results are contrary to the investigation of Mayeuf-Louchart et al. (2017), who observed increased levels of *Atrogin* and *Murf1* mRNA levels in quadriceps muscle of Rev-erb-alpha knockout mice. Stefanetti et al. (2015) did not monitor significant changes for *Atrogin-1* mRNA levels in vastus lateralis muscle of subjects immediately after an endurance or resistance exercise session. Also, the mRNA levels of *Atrogin-1* were increased 2.5, 5.0, and 22 h after the acute endurance exercise.

Here, we showed positive associations between the *Nr1d1* gene with *Ppargc1a*, *Mtor*, and *Bcat2* genes in soleus muscles after a single bout of endurance exercise. Interestingly, most of the alterations of the END group occurred in the predominant oxidative phenotype muscle. It is essential to point out that the gastrocnemius and soleus present phenotypical differences between de muscle fiber composition. For instance, studying BALB/c mice, Dimauro et al. (2019) observed that the myosin heavy chain (MHC)-IIA was the most expressed isoform in gastrocnemius (40.25% ± 6.55) without significant differences among the other isoforms (MHC-IA, 18.6% ± 4.39; MHC-IIX, 6.75% ± 2.75; MHC-IIB, 15.8% ± 3.35). In soleus samples, MHC-IIA was also the isoform more expressed (50.75% ± 4.03), followed by MHC-I (42.20% ± 3.70). Regarding C57BL6J mice, Augusto et al. (2017) observed that the gastrocnemius muscle had (median ± semi amplitude) type IIB (54.42 ± 8.11%), IIDB (19.37 ± 2.98%), IID (2.26 ± 2.24%), IIAD (12.40 ± 2.34%), IIA (5.73 ± 3.24%) and I (5.74 ± 2.55%) fibers. In contrast, the soleus contained type I (37.42 ± 8.20%), IIA (38.62 ± 6.81%), IIAD (18.74 ± 6.95%), and IID (5.69 ± 3.09%) fibers. The authors concluded that the gastrocnemius presented IIB and IIDB fibers predominantly, while the soleus muscles displayed mostly type IIA fibers. Although these fiber type differences may influence the expression of the analyzed genes in response to acute exercise sessions, further investigations should evaluate these genes’ expression in isolated skeletal muscle fibers to confirm this theory.

Altogether, these findings support our initial hypothesis that *Nr1d1* would be increased concomitantly with oxidative capacity-related genes in response to the acute endurance exercise session. In conclusion, an acute bout of endurance exercise elevated the mRNA levels of *Nr1d1* in oxidative skeletal muscle, which was accompanied by an increase of mitochondrial biogenesis and atrophy-related genes. These findings have health implications since the global genetic ablation of this gene was linked to negative adaptations such as the impairment of skeletal muscle oxidative capacity and exercise performance, as well as the increased expression of the atrophy-related genes and reduction of the skeletal muscle fiber size. Future studies should evaluate the circadian rhythm response of *Nr1d1* to different exercise models and the post-translational modifications for metabolism regulation central proteins. Moreover, new studies should assess if the muscle-specific knockout of Nr1d1 blunts these responses to confirm our hypothesis for a mechanistic approach.

## Data Availability Statement

The raw data supporting the conclusions of this article will be made available by the authors, without undue reservation.

## Ethics Statement

The animal study was reviewed and approved by According to the Brazilian College of Animal Experimentation (COBEA), all experimental procedures were approved by the Ethics Committee of the University of São Paulo (I.D 2017.5.33.9037).

## Author Contributions

RR, AR, and AS designed the manuscript. RR and AS wrote the paper. RR, AR, and BM performed the experiments, data collection, and/or statistical analysis. RR designed the figures for the manuscript. AR, RR, BM, JP, DC, ER, and AS contributed to data analysis, discussion, and/or supported financial costs. All authors have read and approved this manuscript.

## Conflict of Interest

The authors declare that the research was conducted in the absence of any commercial or financial relationships that could be construed as a potential conflict of interest.

## Footnotes

1 http://www.genenetwork.org

## References

[B1] Andreux P. A. Williams E. G. Koutnikova H. Houtkooper R. H. Champy M.-F. Henry H. (2012). Systems genetics of metabolism: the use of the BXD murine reference panel for multiscalar integration of traits. *Cell* 150 1287–1299. 10.1016/j.cell.2012.08.012 22939713 PMC3604687

[B2] Augusto V. Padovani C. R. Campos G. E. R. (2017). Skeletal muscle fiber types in C57BL6J mice. *J. Morphol. Sci.* 21:0.

[B3] Bentzinger C. F. Lin S. Romanino K. Castets P. Guridi M. Summermatter S. (2013). Differential response of skeletal muscles to mTORC1 signaling during atrophy and hypertrophy. *Skelet Muscle* 3:6. 10.1186/2044-5040-3-6 23497627 PMC3622636

[B4] Bodine S. C. Stitt T. N. Gonzalez M. Kline W. O. Stover G. L. Bauerlein R. (2001). Akt/mTOR pathway is a crucial regulator of skeletal muscle hypertrophy and can prevent muscle atrophy in vivo. *Nat. Cell Biol.* 3 1014–1019. 10.1038/ncb1101-1014 11715023

[B5] Calvo J. A. Daniels T. G. Wang X. Paul A. Lin J. Spiegelman B. M. (2008). Muscle-specific expression of PPARgamma coactivator-1alpha improves exercise performance and increases peak oxygen uptake. *J. Appl. Physiol.* 104 1304–1312. 10.1152/japplphysiol.01231.2007 18239076

[B6] Da Silva A. S. Pauli J. R. Ropelle E. R. Oliveira A. G. Cintra D. E. De Souza C. T. (2010). Exercise intensity, inflammatory signaling, and insulin resistance in obese rats. *Med. Sci. Sports Exerc.* 42 2180–2188. 10.1249/mss.0b013e3181e45d08 20473230

[B7] Dang K. Li Y. Z. Gong L. C. Xue W. Wang H. P. Goswami N. (2016). Stable atrogin-1 (Fbxo32) and MuRF1 (Trim63) gene expression is involved in the protective mechanism in soleus muscle of hibernating Daurian ground squirrels (Spermophilus dauricus). *Biol Open* 5 62–71. 10.1242/bio.015776 26740574 PMC4728309

[B8] Dimauro I. Antonioni A. Mercatelli N. Grazioli E. Fantini C. Barone R. (2019). The early response of αB-crystallin to a single bout of aerobic exercise in mouse skeletal muscles depends upon fiber oxidative features. *Redox Biol.* 24:101183. 10.1016/j.redox.2019.101183 30974319 PMC6454247

[B9] Egan B. Zierath J. R. (2013). Exercise metabolism and the molecular regulation of skeletal muscle adaptation. *Cell Metab.* 17 162–184. 10.1016/j.cmet.2012.12.012 23395166

[B10] Ferreira J. C. Rolim N. P. Bartholomeu J. B. Gobatto C. A. Kokubun E. Brum P. C. (2007). Maximal lactate steady state in running mice: effect of exercise training. *Clin. Exp. Pharmacol. Physiol.* 34 760–765. 10.1111/j.1440-1681.2007.04635.x 17600553

[B11] Gleeson M. Bishop N. C. Stensel D. J. Lindley M. R. Mastana S. S. Nimmo M. A. (2011). The anti-inflammatory effects of exercise: mechanisms and implications for the prevention and treatment of disease. *Nat. Rev. Immunol.* 11 607–615. 10.1038/nri3041 21818123

[B12] Hardie D. G. Ross F. A. Hawley S. A. (2012). AMPK: a nutrient and energy sensor that maintains energy homeostasis. *Nat. Rev. Mole. Cell Biol.* 13 251–262. 10.1038/nrm3311 22436748 PMC5726489

[B13] Hayasaka M. Tsunekawa H. Yoshinaga M. Murakami T. (2014). Endurance exercise induces REDD 1 expression and transiently decreases mTORC 1 signaling in rat skeletal muscle. *Physiol. Rep.* 2:e12254. 10.14814/phy2.12254 25539833 PMC4332227

[B14] Liu Y. Dong W. Shao J. Wang Y. Zhou M. Sun H. (2017). Branched-chain amino acid negatively regulates KLF15 expression via PI3K-AKT pathway. *Front. Physiol.* 8:853. 10.3389/fphys.2017.00853 29118722 PMC5661165

[B15] Lynch C. J. Kimball S. R. Xu Y. Salzberg A. C. Kawasawa Y. I. (2015). Global deletion of BCATm increases expression of skeletal muscle genes associated with protein turnover. *Physiol. Genomics* 47 569–580. 10.1152/physiolgenomics.00055.2015 26351290 PMC4629004

[B16] Mayeuf-Louchart A. Thorel Q. Delhaye S. Beauchamp J. Duhem C. Danckaert A. (2017). Rev-erb-α regulates atrophy-related genes to control skeletal muscle mass. *Sci. Rep.* 7:14383. 10.1038/s41598-017-14596-2 PMC5662766 29085009

[B17] Popov D. V. (2018). Adaptation of Skeletal Muscles to Contractile Activity of Varying Duration and Intensity: The Role of PGC-1α. *Biochemistry* 83 613–628. 10.1134/s0006297918060019 30195320

[B18] Roberson P. A. Haun C. T. Mobley C. B. Romero M. A. Mumford P. W. Martin J. S. (2018). Skeletal muscle amino acid transporter and BCAT2 expression prior to and following interval running or resistance exercise in mode-specific trained males. *Amino Acids* 50 961–965. 10.1007/s00726-018-2570-2 29725856

[B19] Shimizu N. Yoshikawa N. Ito N. Maruyama T. Suzuki Y. Takeda S. (2011). Crosstalk between glucocorticoid receptor and nutritional sensor mTOR in skeletal muscle. *Cell Metab.* 13 170–182. 10.1016/j.cmet.2011.01.001 21284984

[B20] Stefanetti R. J. Lamon S. Wallace M. Vendelbo M. H. Russell A. P. Vissing K. (2015). Regulation of ubiquitin proteasome pathway molecular markers in response to endurance and resistance exercise and training. *Pflugers Arch.* 467 1523–1537. 10.1007/s00424-014-1587-y 25104573

[B21] Wang H. Sharma N. Arias E. B. Cartee G. D. (2016). Insulin Signaling and Glucose Uptake in the Soleus Muscle of 30-Month-Old Rats After Calorie Restriction With or Without Acute Exercise. *J.Gerontol. A Biol. Sci. Med. Sci.* 71 323–332. 10.1093/gerona/glv142 26341783 PMC5864161

[B22] Wang W. Choi R. H. Solares G. J. Tseng H. M. Ding Z. Kim K. (2015). L-Alanylglutamine inhibits signaling proteins that activate protein degradation, but does not affect proteins that activate protein synthesis after an acute resistance exercise. *Amino Acids* 47 1389–1398. 10.1007/s00726-015-1972-7 25837301

[B23] Williams E. G. Wu Y. Jha P. Dubuis S. Blattmann P. Argmann C. A. (2016). Systems proteomics of liver mitochondria function. *Science* 352:aad0189. 10.1126/science.aad0189 27284200 PMC10859670

[B24] Woldt E. Sebti Y. Solt L. A. Duhem C. Lancel S. Eeckhoute J. (2013). Rev-erb-[alpha] modulates skeletal muscle oxidative capacity by regulating mitochondrial biogenesis and autophagy. *Nat. Med.* 19 1039–1046. 10.1038/nm.3213 23852339 PMC3737409

[B25] Wu Y. Williams E. G. Dubuis S. Mottis A. Jovaisaite V. Houten S. M. (2014). Multilayered genetic and omics dissection of mitochondrial activity in a mouse reference population. *Cell* 158 1415–1430. 10.1016/j.cell.2014.07.039 25215496 PMC4179868

[B26] Yasumoto Y. Nakao R. Oishi K. (2015). Free access to a running-wheel advances the phase of behavioral and physiological circadian rhythms and peripheral molecular clocks in mice. *PLoS One* 10:e0116476. 10.1371/journal.pone.0125646 25615603 PMC4304828

